# Initial examination of the mental health disorders: screening instrument for athletes

**DOI:** 10.3389/fpsyg.2023.1029229

**Published:** 2023-08-04

**Authors:** Brad Donohue, Jesse Scott, Grace Goodwin, Kimberly A. Barchard, Greg Bohall, Daniel N. Allen

**Affiliations:** ^1^Department of Psychology, University of Nevada, Las Vegas, Las Vegas, NV, United States; ^2^The Chicago School of Professional Psychology, Chicago, IL, United States

**Keywords:** sport, athlete, mental health screen, mental health assessment, mental health disorders screening instrument for athletes, referral, clinical utility, factor analysis

## Abstract

**Introduction:**

There is a need to psychometrically develop assessment instruments capable of screening mental health disorders in athlete populations. The current study was conducted to determine reliability, validity and clinical utility of the Mental Health Disorders Screening Instrument for Athletes (MHDSIA).

**Methods and results:**

259 collegiate athletes completed the MHDSIA. Factor analysis determined a single factor with good internal consistency, and this factor was positively correlated with an established measure of psychiatric symptomology (Symptom Checklist 90-R), demonstrating its concurrent validity. An optimum clinical cutoff score (i.e., 32) was determined using Receiver Operating Characteristic (ROC) analyses to assist appropriate mental health referrals.

**Discussion:**

Results suggest the MHSIA is a reliable, valid, and relatively quick and easy to interpret screen for the broad spectrum of mental health disorders in collegiate athletes. As expected, NCAA athletes reported lower MHDSIA scores than club and intramural athletes, while males reported similar severity scores as females.

## Introduction

### Mental health symptomology in collegiate athletes

Annually, in excess of 10,000,000 college students play sports in the U.S. alone (Dugan et al., 2014; NCAA Sport Science Institute, 2016). Although exercise is generally associated with substantial benefits, athletes are potentially exposed to distinct stressors when compared with non-athletes (Arnold and Fletcher, 2012); which increases their risk of developing mental health-related problems (Sudano and Miles, 2017). While the actual prevalence of psychological problems within athletes has been debated, the notion that athletes are protected against mental health problems has been shown to be inaccurate (Wolanin et al., 2015). The rates of psychological disorders found in student-athletes and non-athletes tends to be very similar. Approximately 20% of adults suffer from significant mental-health problems on an annual basis (Hedden et al., 2015). However, the emergence of social media has created a platform for athletes to be publicly criticized (Kristiansen et al., 2012), and pressure to win competitions, entertain fans, and please coaches and families can lead to chronic feelings of stress, as athletes tend to display depressive symptoms after failing to achieve goals (Hammond et al., 2013) or losing competitions (Jones and Sheffield, 2008). The emerging adult years are associated with peak athletic performance, and are the years athletes are at highest risk for the onset of mental health disorders (Gulliver et al., 2012). Financial concerns, travel commitments, and maintaining academic eligibility requirements are common stressors within the student-athlete population (Rice et al., 2016). Student-athletes are expected to be successful in their classes, just as other students, but they are also expected to excel in athletic competition. Athletes often experience both physical and mental fatigue and report feeling socially isolated (Parham, 1993). In many cases, athletes must maintain superior levels of physical fitness and adhere to excessive sport-related time demands (Broughton and Neyer, 2001). There is evidence showing the daily stress associated with athletic participation may contribute to depression (Serido et al., 2004). Athletes who are subject to extreme training regimens tend to report higher levels of stress and depression than those who train less (Nixdorf et al., 2015), and intense physical activity has been shown to compromise physical well-being and increase symptoms of depression and anxiety in athletes, often due to injury, overtraining, or burnout (Peluso and Andrade, 2005). Meeusen et al. (2013) found that overtraining combined with insufficient recovery time may elevate athletes’ risk of developing a psychological disorder.

Student-athletes often report personal relationship difficulties, in addition to reduced energy and, and low levels of motivation (Parham, 1993). Living in an unfamiliar environment, reduced support networks due to relocation, and poor adjustment to life after sports are all associated with depression in athletes (Fletcher and Wagstaff, 2009). Over 40% of athletes in one study demonstrated low quantity of sleep and/or the quality of their sleep was poor (Mah et al., 2018). Chronically restricted sleep has important implications for potential injury (Luke et al., 2011), susceptibility to infectious illness (Prather et al., 2015), and the accuracy of concussion assessment (Silverberg et al., 2016). Additionally, restricted sleep has been shown to reduce reaction times (Dinges et al., 1997) and impair sport execution (Reyner and Horne, 2013), which both may have a negative impact on the athletes’ performance and, subsequently, their overall mental health.

The intense mental and physical demands of sport participation have been associated with risky behaviors (Hughes and Leavey, 2012). Nearly half of collegiate athletes are likely to experience an injury preventing them from participating in their sport, for at least a brief period of time (Meeuwisse et al., 2003), and injuries have been shown to increase athlete vulnerability to a variety of mental illnesses (Gulliver et al., 2015).

Investigations into the mental health of sub-groups of athletes are exceedingly rare. Researchers in France found female elite athletes were 1.3 times more likely to receive a diagnosis of a mental health disorder as compared to male athletes (Schaal et al., 2011). Many athletes display depression or anxiety related to adjusting to life outside of sports after their playing careers are over (Giannone et al., 2017). As compared to the general population, retired male athletes from team sports have consistently endorsed higher rates of distressing psychological symptoms (Van Ramele et al., 2017). Even athletes simply contemplating or preparing for retirement have shown elevations in self-reported symptoms of depression than those who are not considering retirement (Beable et al., 2017).

Evidence suggests high-level athletes demonstrate a similar risk of developing anxiety disorders as compared to the general population (Gulliver et al., 2015). Similarly, 17% to 21% of college-aged males and females meet criteria for depression in both athlete and non-athlete populations (Yang et al., 2007; Weigand et al., 2013) and nearly one-quarter of females in this age group, including athletes, endorse subthreshold symptoms of disordered eating (Greenleaf et al., 2009). One of the key concerns for mental health providers who treat college athletes is the high rate of comorbidity of psychological problems in this population. For example, student-athletes who demonstrate symptoms of eating disorders are likely to endorse elevated anxiety levels (Vardar et al., 2007), and athletes who endorse symptoms of depression display higher rates of alcohol abuse and dependence (Miller et al., 2002).

Depression is a major contributor to preventable hospitalizations (Davydow et al., 2015), associated with higher rates of substance use disorders, and linked with increased suicide risk [U.S. Department of Health and Human Services, National Institutes of Health, National Institute of Mental Health (NIMH), 2015]. In another study involving more than 200 athletes, almost half reported symptoms of depression or anxiety (Gulliver et al., 2015).

For providers in the most frequent contact with student-athletes, monitoring athlete mental health should be treated with the utmost importance, as the relationship between mental and physical health has been demonstrated repeatedly (Yang et al., 2014). For example, student-athletes experiencing symptoms of depression are more likely to exhibit a decline in sport performance or to suffer a sport-related injury. The same injury risk and performance-related problems have been linked to athletes who are diagnosed with eating disorders or alcohol abuse (Mountjoy et al., 2014).

Between 2008 and 2012, nearly one-third of the males and half of the females who participated in National Collegiate Athletic Association (NCAA) sports endorsed experiencing depressive or anxiety-related symptoms (Brown et al., 2014). Substance use is also a significant problem in university athletic populations (Donohue et al., 2018). In addition, student-athletes demonstrate an elevated risk of developing sleep problems (Brown et al., 2014), and disordered eating (Bratland-Sanda and Sundgot-Borgen, 2013.

Student-athletes have repeatedly demonstrated similar rates of clinical depression (Yang et al., 2007; Weigand et al., 2013) and eating disorders (Greenleaf et al., 2009), when compared to non-athlete peers. However, some findings suggest athletes may be particularly susceptible to these two disorders (Rice et al., 2016). It is possible certain subgroups of athletes demonstrate an elevated risk for eating disorders. For example, athletes who need a lean body shape to compete in their particular sport and female athletes in a variety of sports tend to be at higher risk than individuals in the general population (Byrne and Mclean, 2002; Sundgot-Borgen and Torstveit, 2010).

Athletes have also demonstrated a higher rate of alcohol use than the general population in some studies, possibly due to a binge pattern of consumption during the offseason or vacations (Mastroleo et al., 2018). Student-athletes report more problematic alcohol use and riskier drinking patterns than non-athletes, which could be related to the elevated physiological and psychological stress associated with sport participation (Brenner and Swanik, 2007). Male athletes as well as Caucasian athletes have been shown to drink alcohol more excessively than other groups (Leichliter et al., 1998). Although, this may not be unique to athletes as these same factors have been shown to be more predictive of drinking levels among college students in general (Baer, 2002). In another study, weekly alcohol use doubled the risk of injury among participants (O’Brien and Lyons, 2000).

There is some evidence to suggest male athletes use more illicit substances than their female counterparts; yet, rates of self-reported illicit drug use are typically low within the elite athletic population (Buckman et al., 2013). In a study examining the drug and alcohol use of over 1,000 student-athletes at Division I schools, over one-third of respondents endorsed taking banned performance-enhancing drugs, but less than 5 percent of them said they would disclose their use of drugs to coaches or other health care providers. Similarly, almost half of the athletes endorsed drinking more than five drinks in a week, yet only 3 percent said they would openly admit to doing so (Druckman et al., 2015). This provides some evidence that self-underreporting may be a problem when it comes to the assessment of drug and alcohol use among college athletes (Buckman et al., 2013). In a treatment outcome study requiring collegiate student athletes to evidence alcohol or illicit substance use in the past 4 months, more than 50% evidenced a current mental health disorder and 80% evidenced a current or past mental health disorder, most often a substance use disorder (Donohue et al., 2018).

### Challenges in the identification of mental health symptomology in athletes

Most universities are ill-equipped to assess the psychological concerns of student-athletes due to a lack of athlete-specific assessment tools (Donohue et al., 2019; Hussey et al., 2019). Additionally, universities that do offer mental health screening options use screens that are difficult to interpret for those who are likely to administer these instruments (Watson, 2006). Identifying psychological disorders, especially in the case of subthreshold presentations, is a difficult undertaking for individuals who are not trained in mental health. One major barrier to the identification of mental health issues by untrained providers is that it can be challenging to differentiate between common athlete behaviors and symptoms of psychological disorders (Sundgot-Borgen and Torstveit, 2010). For instance, dedicated athletes may adhere to strict eating and exercise regimens that may appear to be disordered eating behaviors (Thompson and Sherman, 1999). Similarly, many athletes complain of fatigue, which could be a natural result of excessive sport training or, potentially, a symptom of depression (Esfandiari et al., 2011). Concussions have also been found to produce a negative impact on a student-athletes’ cognitive functioning for an extended period of time (Thoma et al., 2015). Concussion-related cognitive issues may be difficult to distinguish from poor concentration, which is a common symptom of multiple mental health disorders. Anxiety related to athletic performance, maintaining eligibility requirements, or lack of playing time may be challenging to discern from generalized anxiety. In difficult cases like these, a screening tool that aids in the early detection of potential psychological problems could differentiate between athletes who may benefit from mental health treatment and those who are not currently in need of services. Although, screening measures are not sufficient to diagnose mental health disorders, identifying dysfunction beyond what might be expected in sport settings can be the first step toward diagnosis and effective intervention.

### Variation in assessment resources/practices in the United States

The mental health resources provided by universities for student-athletes vary significantly across the United States (Gallagher, 2012). Schools with larger athletic departments (i.e., Division I) tend to have larger mental health budgets than smaller schools (i.e., Division II or III; Matheson et al., 2012) and are more likely to have comprehensive athlete mental health screening programs (Kroshus, 2016). However, even in Division I programs, less than one-third of schools require their athletes to participate in annual mental health screening (Watson, 2006) and many universities fail to implement mental health screening of athletes altogether, possibly due to a shortage of qualified mental health providers or, perhaps more importantly, a lack of efficient and validated screening measures (Matheson et al., 2012). In a survey including 127 head athletic trainers of NCAA Division I schools, it was found only 42.5% of those schools utilized a mental health-screening instrument (Sudano and Miles, 2017). Moreover, the instruments currently in use do not assess mental health symptoms or these instruments have not been validated within athlete populations (Kroshus, 2016). The predominate mental health assessments currently in use include the Patient Health Questionnaire (PHQ-9; Kroenke et al., 2001), the Generalized Anxiety Disorder (GAD-7; Spitzer et al., 2006), Symptom Checklist 90 revised (SCL-90-R; Derogatis, 1994), and the Pre-Participation Physical Examination (PPE; Smith and Laskowski, 1998). Although these assessment measures have been psychometrically validated in non-athlete populations (Derogatis, 1994; Kroenke et al., 2001; Martin et al., 2006), in the case of the PHQ-9 and GAD-7 respectively, they only offer an assessment of one mental health issue (e.g., depression or anxiety) and neglect to examine the wide range of possible mental health concerns faced by athletes. The PPE is widely used to screen athletes for physical or medical concerns (i.e., respiratory problems, vision, pre-existing health conditions), but does not assess for the presence of psychological issues (Cottone, 1999). The Symptom Checklist 90-Revised (SCL-90-R; Derogatis, 1994) is currently one of the most frequently implemented tools for assessing global psychological symptomatology in non-athlete populations. The SCL-90-R is a 90-item questionnaire designed to assess nine distinct dimensions of mental health functioning. The SCL-90-R’s reliability and validity have also been examined in multicultural settings (Martin et al., 2006). This measure has also been utilized to examine the psychometric properties of a wide variety of screening instruments (Øiesvold et al., 2011). However, its length may make it less economical to administer.

Compounding the problem of inadequate screening, there is reason to believe athletes’ responses on these measures may not be accurate, perhaps because athletes tend to be unaware of the relationship between athletic performance and mental health (Buckman et al., 2013; Neal et al., 2013), and because they may be concerned with potential negative consequences for reporting mental health dysfunction. Due to this lack of awareness, student-athletes are considered a unique population for which sport-focused mental health assessment may be more effective (Comeaux et al., 2017). There are three sport-specific mental health screens. The Sport Interference Checklist (*SIC*; Donohue et al., 2007) is a validated measure that identifies mental health interferences in sport competition and training. Examinations of the *SIC* have demonstrated its strong factor structure and concurrent validity (Donohue et al., 2007), and it is capable of reliably screening symptoms of mental health disorders in athletes (Donohue et al., 2018). However, it does not assess the extent to which mental health symptoms may interfere in life outside of sports. The Student Athlete Relationship Inventory (SARI; Donohue et al., 2007) is another reliable and valid sport-specific method of screening mental health in athletes (Donohue et al., 2007). Its items assess sport-specific problems experienced by athletes with their coaches, teammates, family. However, SARI item stems are not directly relevant to mental health symptoms, thus it lacks face validity. Lastly, the Athlete Psychological Strain Questionnaire (APSQ; Rice et al., 2020) is a 10 item screening instrument for mental health problems, and includes 3 subscales (i.e., Self-regulation, Performance, External Coping). This scale has demonstrated excellent reliability and validity in elite athletes. Although these measures have successfully identified athletes who are at-risk for mental health treatment, the item stems do not explicitly assess the wide array of mental health disorders.

Understaffing and lack of financial resources make effective screening tools important in university athletic systems, particularly in smaller schools, with presumably lower budgets allocated to athlete mental health. Some have advocated for the utilization of brief validated screening measures that do not require professional interpretation (Eisenberg et al., 2007). Identifying psychological disorders may be profoundly impactful in addressing the mounting financial strain on colleges and athletic departments. Consequently, interest is rising among university administrations to improve mental health screening instruments; thus, reducing long-term mental health care costs (Neal et al., 2013; Rao et al., 2015; Wolanin et al., 2015).

A major obstacle in connecting athletes with mental health services is the lack of mental health assessment tools that have been validated in athlete populations, are psychometrically valid, and are capable of identifying both clinical and subclinical mental health symptomology. A measure examined explicitly in athletes, easy to administer, and that reliably assesses mental health problems could help better assist athletes in their receipt of mental health services when needed. The aforementioned sport-specific screens do not comprehensively and explicitly assess mental symptomology factors outside of sport (Bar and Markser, 2013). Indeed, while it is important for student athletes to perform well in sport settings, stakeholders of athletes should also work to ensure student-athletes are able to perform well in their life outside of sport (Lazarus, 2000).

Importantly, investigators have yet to examine how severity of athletes’ mental health symptomology is related to their performance in life outside of sports. This is an important oversight given the increased interest sport organizations have expressed in treating athletes holistically. Along these lines, existing sport-specific mental health assessments have demonstrated reliability and validity in athlete samples, and the factor scores of these items have been shown to accurately predict mental health symptomology within sport settings. However, these scales do not explicitly assess the wide range of mental health disorders that are evidenced in athlete populations.

The purpose of the present study was to psychometrically examine the utility of a novel mental health screening measure in a sample of collegiate athletes (i.e., Mental Health Disorders Screening Instrument for Athletes; MHDSIA), including an evaluation of this measure’s factor structure, internal consistency, convergent validity, and clinical cutoff scores to assist in determining collegiate athletes who may benefit from mental health referrals.

## Methods

### Participants

Participants were 259 undergraduate students enrolled at a National Collegiate Athletic Association (NCAA) Division I university in the United States. Participants were at least 18 years of age; competed in NCAA, club, or intramural sports; consumed at least one alcoholic drink or non-prescribed drug during the previous 4 months according to self-report, and expressed interest in receiving goal-oriented psychologically-based intervention in an outcome study funded by the National Institutes of Health (NIH) in the United States.

### Measures

#### Mental health disorders screening instrument for athletes

Eight collegiate students with competitive sport experience created the measure in a focus group with a licensed clinical psychologist who acted as moderator. The goal was to create one inventory specific to mental health factors, with each item stem encapsulating a mental health disorder in the DSM-5. The moderator facilitated discussion (Krueger and Casey, 2000), while maintaining minimal control over discussion content, consistent with Millward’s (1995) approach. The moderator’s other main responsibility was to ensure efficiency and depth of discussion. Item stems were derived to fit within the following context: “How often does [item stem] interfere with performance during life outside of sports?” This was conceptually important because in the DSM-5 almost all mental health disorders require functional impairment.

Symptom frequency has proven to be a reliable indicator of functional impairment; thus, it was determined that a frequency response format would be appropriate for this measure. According to Weng (2004), a 7-point frequency scale is the preferred response scale in college student samples. Consequently, a 7-point frequency scale was selected for this measure, ranging from 1 (never) to 7 (always). Specifically, the athletes are asked to respond how often the item content interferes with performance outside of sports. A copy of this scale is provided in Appendix.

#### Symptom checklist 90-revised

The Symptom checklist 90-revised (SCL-90-R; Derogatis, 1994) is a 90-item self-report measure, assessing nine distinct dimensions of mental health functioning. The SCL-90-R prompts participants to rate the severity of 90 symptoms over the past week using a 5-point scale from 0 to 4, on which a 0 indicates the participant has not experienced the symptom in the past week and a 4 indicates the participant has been extremely distressed by the symptom over the past week. The Global Severity Index (GSI) is this measure’s assessment of general psychological impairment. The GSI has been shown to have strong psychometric properties in a number of studies (Derogatis, 1994). The GSI was used in the present study to evaluate whether general psychological impairment is associated with psychological interference with the athletes’ performance in their lives outside of their respective sport. In previous studies, both college athletes at various levels of competition (i.e., NCAA, recreational) have exhibited significantly lower GSI scores as compared to their non-athlete peers (Donohue et al., 2004).

### Procedure

The current study occurred within the context of baseline assessment in a randomized clinical trial. Participants in this study were recruited to determine their interest in being randomized to one of two goal-oriented programs (i.e., traditional campus counseling and psychological services, The Optimum Performance Program in Sports; see Donohue et al., 2018). Participants were recruited in several ways, including course credit, Athletics Department referral consequent to problematic alcohol use or illicit drug use, flyers describing the study that were circulated throughout campus, after study presentations during athletic team meetings and performance workshops. After study consent, participants completed study measures. The study was approved by the university’s institutional review board (IRB; #1206-4,177 M). No adverse events occurred during the course of this study. The participants completed the study within the context of a certificate of confidentiality issued by the NIH, which protects the participants’ records from being released due to court mandate.

### Study hypotheses

*H1:* Based on the results of factor analysis, each item from the experimental measure will load onto a single factor because all items are psychiatric constructs impacting life performance outside of sports.

*H2:* Items within the resulting factor will evidence high internal consistency.

*H3:* The hypothesized factor score (MHDSIA) will evidence a positive correlation with the SCL-90-R Global Severity Index (GSI), demonstrating its concurrent validity.

*H4:* Males will demonstrate significantly lower scores on the MHDSIA and GSI, as compared with females.

*H5:* NCAA athletes will demonstrate lower scores on the MHDSIA and GSI than club and intramural athletes.

*H6:* A cutoff score will distinguish between non-clinical and clinical level mental health concerns.

### Statistical analysis

Principal component analysis will be performed on the MHDSIA inventory. The number of factors will be established according to the results of parallel analysis (Horn, 1965) and Minimum Average Partial (MAP, Velicer, 1974) tests. A sample size of 100 to 200 participants has been shown to produce a reasonably stable factor structure, although 300 participants are recommended (Guadagnoli and Velicer, 1988).

Cronbach’s alpha will be applied to calculate the internal consistency of all resulting factors. Feldt’s (1965) method of calculating confidence intervals for Cronbach’s alpha will be used to calculate confidence intervals for ICC (A,k). Total scores will be calculated for all recognized factors to establish a numerical representation of each participant’s total pattern of symptomology. Participants’ total scale scores will be calculated by summing their responses to items in the experimental measure. Correlations will also be calculated between the MHDSIA and SCL-90-R Global Severity Index (GSI) to determine the convergent validity of the measure. A positive correlation is expected between the scores of the experimental measure and the SCL-90-R GSI because mental health symptom severity would presumably be associated with an increase in psychological interference outside of sports.

To determine if participants of different genders and varying sport levels exhibited similar response patterns on the MHDSIA and SCL-90-R, a multivariate analyses of variance (MANOVA) will be performed using the MHDSIA and SCL-90-R as dependent variables and athlete type (NCAA, club, recreational) as the independent variable, and a similar MANOVA will be performed with gender as the independent variable. Post-hoc analyses will be performed for significant MANOVAs.

To explore whether the MHDSIA scale can be used to assist in the referral of athletes to mental health services, Receiver Operating Characteristic (ROC) analyses will be used to predict MHDSIA scores that are significantly associated with clinically elevated scores on the SCL-90-R GSI. ROC analysis will be used to generate empirical cut-off scores for the MHDSIA. Factor scores on the MHDSIA corresponding to a T score of 60 on the SCL-90-R GSI will be considered clinically relevant and can be considered cues for referral to appropriate mental health services. Cut off scores will be identified using Youden’s Index, which equals sensitivity + specificity – 1, and thus maximizes overall correct classification.

## Results

### Participant demographic and assessment variables

Table 1 displays the participants’ demographic information and Table 2 displays the means and standard deviations for the primary study variables.

**Table 1:** Demographic information (*N* = 259).

Item	Total	%
**Gender**		
Male	144	55.6
Female	115	44.4
**Ethnicity**		
White/Caucasian	102	39.4
Black/African-American	39	15.1
Asian/Asian American	23	8.9
Hispanic/Latino	32	12.4
Pacific Islander	11	4.2
Other (multiple or not listed)	52	20.1
**Type of athlete**		
NCAA	102	39.8
Club	38	14.7
Intramural	119	46.0
**Class status**		
Freshman	105	40.5
Sophomore	75	29.0
Junior	53	20.5
Senior	26	10.0

Average age 19.83 (SD = 2.06).

**Table 2:** Means and standard deviations of study variables (*n* = 259).

Measures	M	SD	Min	Max
MHDSIA	30.08	12.31	14.00	89.00
SCL-90-R GSI	0.62	0.43	0.00	2.22

### Outliers

Mahalanobis distance analysis indicated the associated for value of p for 257 of the sets was greater than 0.001 and, therefore, these sets are not considered outliers (Tabachnick and Fidell, 2013). Two sets of scores did meet the 0.001 alpha outlier criterion; however, after examination of the scores it was determined these extreme scores were likely not due to error and both indicated fewer self-identified mental health symptoms endorsed on the SCL-90-R as compared to the experimental measure. Due to the minimal number of outliers and the expected variability within a sample of this size, no cases were selected for removal.

### MHDSIA

A principal components analysis revealed a one-factor solution for the MHDSIA, comprising 36.2% of the total variance. As displayed in Table 3, items loaded onto one factor related to general mental health symptoms. The scale items demonstrated high internal consistency (Cronbach’s α = 0.86, 95% CI [0.83, 0.88]) and standardized alpha (0.86). The intraclass correlation for absolute agreement [ICC (A,k)] was also strong (0.83, 95% CI [0.79, 0.86]). Corrected item-total correlations and alphas-if-item-deleted were also performed on the items of the MHDSIA. The results are summarized in Table 4. As no corrected item-total correlations were below 0.3, the deletion of any items would not improve internal consistency. All alpha-if-item-deleted values fell between 0.84 and 0.85. As the coefficient alpha of the 14-item measure is 0.86, the removal of any items would not improve internal consistency of this measure. Convergent validity for the MHDSIA was assessed by correlating the total factor score with the SCL-90-R Global severity index (GSI). The correlation between this scale and the GSI was significant (*r*(25) = 0.62, *p <* 0.008, 95% CI [0.53, 0.68]) and demonstrates a strong positive association between the two measures, which indicates that as athletes experience an increase in mental health symptoms, they experience related interference with their lives outside of sport.

**Table 3:** First principal component (MHDSIA).

Item	Pattern matrix coefficient
1. Too impulsive	0.48
2. Feeling depressed	0.69
3. Severe anxiety, panic attacks, obsessive thoughts, doing senseless behavior repeatedly	0.67
4. Alcohol use	0.62
5. Drug use, or use of prescribed drugs more than medical doctor’s recommendation	0.52
6. Difficulty maintaining weight at an acceptable level to me or to others	0.48
7. Difficulty sleeping	0.55
8. Doing things that get me in trouble with others	0.67
9. Poor relationships with others	0.68
10. Tics or sudden and uncontrollable jerks of body parts	0.52
11. Hearing, smelling, or seeing things that others do not	0.51
12. Difficulties remembering things	0.62
13. Sudden mood swings	0.69
14. Sexual disorders (pain during sex, premature ejaculation, problems with arousal, sexual promiscuity, unsafe sex)	0.66

Coefficient alpha for the first principal component is 0.86.

**Table 4:** Item-total statistics (MHDSIA).

Item	Corrected item-total correlation	Coefficient alpha if item deleted
1. Too impulsive	0.40	0.85
2. Feeling depressed	0.63	0.84
3. Severe anxiety, panic attacks, obsessive thoughts, doing senseless behavior repeatedly	0.59	0.84
4. Alcohol use	0.53	0.84
5. Drug use, or use of prescribed drugs more than medical doctor’s recommendation	0.43	0.85
6. Difficulty maintaining weight at an acceptable level to me or to others	0.41	0.85
7. Difficulty sleeping	0.48	0.85
8. Doing things that get me in trouble with others	0.58	0.84
9. Poor relationships with others	0.60	0.84
10. Tics or sudden and uncontrollable jerks of body parts	0.41	0.85
11. Hearing, smelling, or seeing things that others do not	0.40	0.85
12. Difficulties remembering things	0.53	0.84
13. Sudden mood swings	0.61	0.84
14. Sexual disorders (pain during sex, premature ejaculation, problems with arousal, sexual promiscuity, unsafe sex)	0.55	0.84

Coefficient alpha for the 14-item test is 0.86.

### Analysis of potential effects due to gender and athlete type

#### Gender

It was predicted that males would exhibit more severe mental health symptomology on the MHDSIA and SCL-90R (see Table 5 for means and standard deviations). To examine this hypothesis, a MANOVA was performed utilizing the MHDSIA and the SCL-90-R as dependent variables. There was a statistically significant difference in gender, *F*(2, 256) = 3.73, *p* < 0.05, Wilk’s Λ = 0.972. To determine the source of these differences, one-way ANOVAs were performed using the MHDSIA scale and the SCL-90-R as dependent variables and gender as the independent variable for analyses. There were no significant differences between genders on the MHDSIA scale [*F*(1,257) = 0.004, *p* > 0.05]; however, there was a difference between genders on the SCL-90-R [*F*(1,257) = 4.69, *p* < 0.05]. Specifically, female athletes endorsed the presence of more overall mental health symptoms than male athletes, *p* < 0.05.

**Table 5:** Screening measure scores across gender and sport level.

Measure	Total Sample (*N* = 259)		Male (*n* = 144)		Female (*n* = 115)		NCAA (*n* = 102)		Club (*n* = 38)		Intramural (*n* = 119)	
	*M*	*SD*	*M*	*SD*	*M*	*SD*	*M*	*SD*	*M*	*SD*	*M*	*SD*
MHDSIA	30.08	12.31	30.04	13.42	30.14	10.82	27.31	11.03	33.39	12.61	31.40	12.87
GSI	0.62	0.43	0.57	0.41	0.68	0.44	0.54	0.43	0.78	0.54	0.63	0.38

NCAA, National Collegiate Athletic Association; GSI, SCL-90-R Global Severity Index; MHDSIA scores are reported as raw scores; GSI scores are reported as T-scores.

#### Athlete type

It was also expected that different types of athletes (intramural, club, NCAA) would exhibit similar response patterns on the MHDSIA and SCL-90-R (see means and standard deviations for these scale in Table 5). To test this hypothesis, a MANOVA was performed utilizing the MHDSIA total score and the SCL-90-R GSI as dependent variables and sport type as the independent variable. There was a statistically significant difference in responses based on whether the participant was an NCAA, club, or intramural athlete *F*(4, 508) = 3.27, *p* < 0.005, Wilk’s Λ = 0.95. To determine the source of these differences, one-way ANOVAs were performed using the MHDSIA scale and the SCL-90-R as dependent variables and sport type as the independent variable for each analysis.

There were significant differences between athlete types on the MHDSIA scale [*F*(2,255) = 5.37, *p* < 0.01] and the SCL-90-R [*F*(2,255) = 8.40, *p* < 0.01]. A Tukey *post hoc* test revealed that NCAA athletes scores on the MHDSIA were significantly lower than club athletes and intramural athletes. There was a statistically significant difference between groups on the SCL-90-R scores [*F*(2,255) = 4.613, *p* < 0.05]. A Tukey *post hoc* test revealed that NCAA athletes responded significantly lower than club athletes on this scale (*p* < 0.01).

#### ROC results

**Figure 1:**
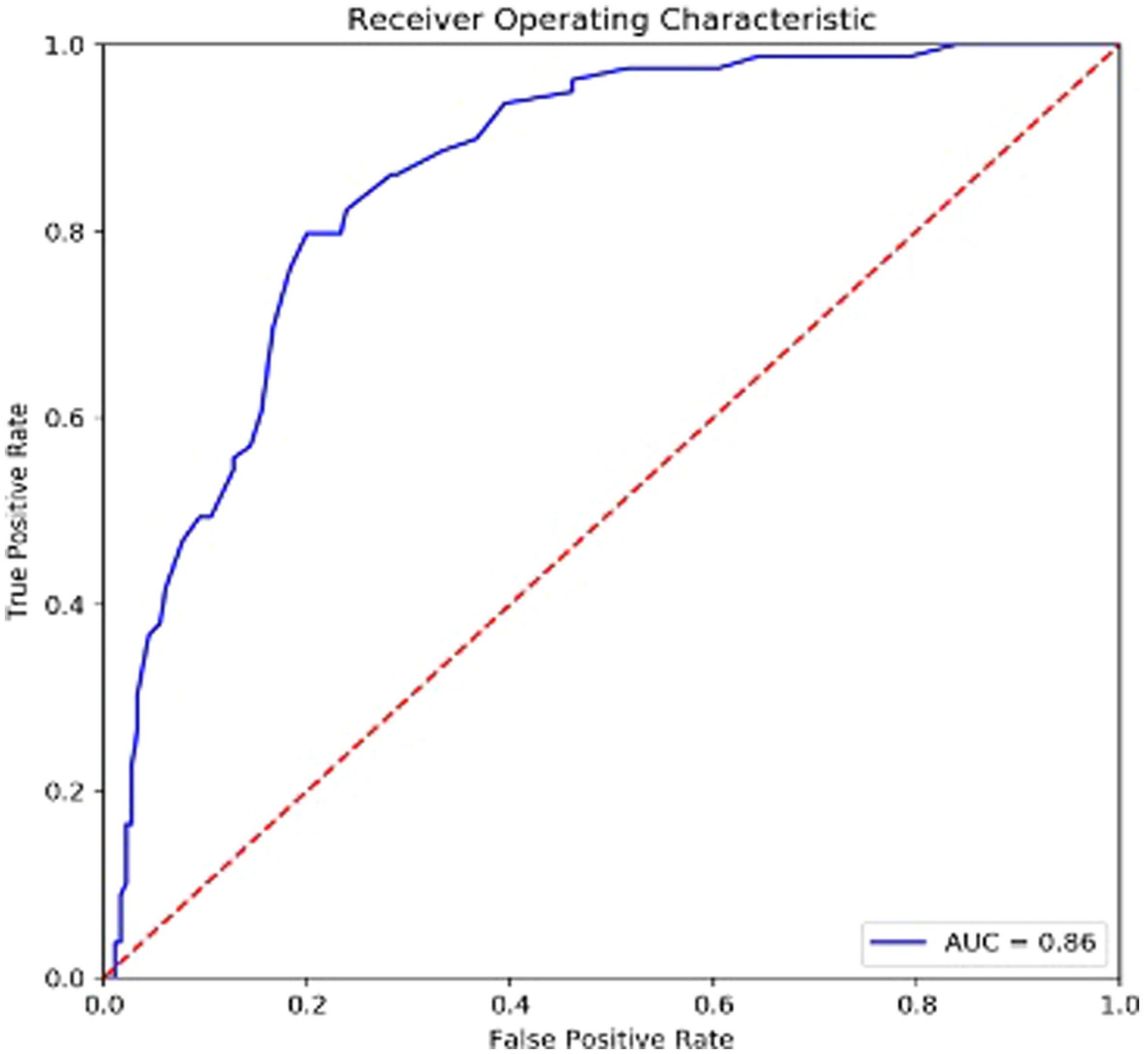
ROC curve for MHDSIA.

For the ROC analysis, athletes were divided into two groups, in which 80 athletes demonstrated subclinical to clinical levels of mental health concerns (T-scores > 60) and 179 athletes demonstrated nonclinical levels of concern (T-scores < 60). For the MHDSIA, the variances in sensitivity, specificity, positive predictive value (PPV), and negative predictive value (NPV) were evaluated. The PPV and NPV are accuracy statistics that indicate the number of identified positive cases that actually are of High Risk vs. those that are actually of Low Risk (compared to false positive and false negative classifications; Stojanovic et al., 2014). The MHDSIA’s ability to distinguish between athletes of High and Low-Risk for mental health difficulties was measured using the Area under the ROC curve (AUC), with an AUC of 0.50 indicating chance classification, and AUC of 1.00 indicating a perfect classification rate (Hosmer et al., 2013). AUCs were compared using the method of Hanley and McNeil (1983). Table 6 presents details on the AUCs for the MHDSIA. Cut off scores were ascertained using Youden’s Index (sensitivity + specificity – 1), thus, maximizing overall correct classification. Figure 1 presents the ROC curve for the MHDSIA, and Table 7 presents sensitivity, specificity, PPV, NPV, correct classifications, and diagnostic likelihood ratios (DLR) for each analysis. Results indicate that the MHDSIA identified High Risk athletes significantly better than chance, with an AUC of 0.86.

**Table 6:** Receiver operating characteristic (ROC) area under the curve (AUC) for the MHDSIA for classification of SCL-90-R global severity index scores.

	AUC	95% CI of AUC	SE of AUC	p*
MHDSIA	0.86	0.81–0.90	0.02	<0.001

MHDSIA, Sport Interference Checklist Life Outside of Sports Inventory; SE, Standard error; AUC, Area under the curve. *indicates asymptotic significance level.

**Table 7:** Classification accuracy statistics for SCL-90-R global severity index.

	Score^a^	TP	FP	TN	FN	Sn	Sp	PPV	NPV	DLR^b^
MHDSIA	14	79	180	0	0	1	0	0.31	0	1
	19	78	143	37	1	0.99	0.21	0.35	0.97	1.24
	24	77	93	87	2	0.97	0.48	0.45	0.98	1.89
	26	74	71	109	5	0.94	0.61	0.51	0.96	2.37
	28	70	60	120	9	0.89	0.67	0.54	0.93	2.66
	30	65	43	137	14	0.82	0.76	0.60	0.91	3.44
	**32**	**63**	**36**	**144**	**16**	**0.80**	**0.80**	**0.64**	**0.90**	**3.99**
	34	55	30	150	24	0.70	0.83	0.65	0.86	4.18
	36	45	26	154	34	0.57	0.86	0.63	0.82	3.94
	40	37	14	166	42	0.47	0.92	0.73	0.80	6.02
	46	18	5	175	61	0.23	0.97	0.78	0.74	8.20
	54	8	4	176	71	0.10	0.98	0.67	0.71	4.56

TP, number of true positive classifications; FP, number of false positive classifications; TN, number of true negative classifications; FN, number of false negative classifications; Sn, Sensitivity; Sp, Specificity; PPV, Positive Predictive Value; NPV, Negative Predictive Value; MHDSIA, Sport Interference Checklist Life Outside of Sports Inventory; MHDSIA Total Score can range from 14 to 98; DLR, diagnostic likelihood ratio.

a Bolded scores represent the optimal cut score as determined by Youden’s Index. ^b^The probability of a high-risk athlete being correctly classified into the high-risk group for overall mental health symptoms.

Given that NCAA and Club athletes had significantly different mean scores on the SCL-90-R and theMHDSIA, ROC analyses were performed on each athlete group separately to determine whether a cutoff score of 32 would be appropriate across these groups. The results of each ROC analysis indicated that a cutoff score of 32 is optimal regardless of athlete type. Indeed, in the case of NCAA and Club athletes, sensitivity and specificity both increase when groups are examined independently. For NCAA athletes, a cutoff score of 32 produces a sensitivity of 0.85 and a sensitivity of 0.91, while the AUC increases to 0.94. For Club athletes, a cutoff score of 32 produces a sensitivity of 0.88 and a specificity of 0.86, while the AUC increases to 0.96. Lastly, for intramural athletes, a cutoff score of 32 offers the highest diagnostic likelihood ratio, although sensitivity decreases to 0.72 and specificity decreases to 0.69.

## Discussion

There is a great need to empirically develop mental health screening tools in athlete samples. Indeed, the extant measures that have been evaluated in the assessment of athletes’ mental health tend to be focused on the impact of psychological issues experienced during sport performance competition or training (Donohue et al., 2018, 2019; Hussey et al., 2019; Rice et al., 2019, 2020), potentially neglecting assessment of symptoms manifesting in athletes’ lives outside of sports. Along this vein, mental health-related problems outside of sport must be considered carefully when working with student-athletes. According to the APA (2013), mental health disorders are often marked by functional impairments in multiple settings. In the assessment of athletes’ mental health, a decline in sport performance due to outside stressors is relatively common. However, these stressors are often not openly discussed with team physicians or coaches (Reardon and Factor, 2010). Therefore, assessing athletes’ mental health symptomology as an interference with performance, as compared with a pathological framework (i.e., dysfunction), may be an effective strategy to improve identification of treatment targets by reducing potential stigma. The current study was designed to examine the factor structure, reliability, and validity of a mental health screening measure explicitly for use in collegiate athlete populations (i.e., MHDSIA).

Results indicated that each individual item on the MHDSIA evidenced a salient loading on a single factor, and the MHDSIA demonstrated strong convergent validity with the a widely established measure of psychiatric symptomology (i.e., SCL-90-R GSI), as predicted. Although many items included in the MHDSIA overlap with items in the SCL-90-R (e.g., depressed mood, anxiety), the MHDSIA offers unique advantages. Indeed, the MHDSIA is relatively brief, and its items prompt athletes to explore how the respective content impacts functioning outside of sports. Males endorsed significantly fewer items on the SCL-90-R than females but did not demonstrate significant differences from females in their responses to the MHDSIA. This is an important finding due to the tendency of males to endorse fewer psychological symptoms than females (Grant et al., 2005) due, in part, to men having greater perceptions of stigma associated with reporting mental health symptoms. Thus it is possible the MHDSIA may have lowered perceived mental health stigma that is often found in male athlete populations.

MHDSIA scores differentiated elite athletes (i.e., NCAA) from club and intramural athletes, providing support for its discriminate validity. This is an important finding as athletes competing at various levels of competition have demonstrated similar psychiatric symptomology (Donohue et al., 2004), and have evidenced similar psychiatric improvements subsequent to mental health treatment (Donohue et al., 2018). Nevertheless, examining MHDSIA responses of NCAA, intramural, and club athlete groups separately may be an area of focus in future research.

ROC analysis indicated the MHDSIA is an effective tool for detecting athletes who may be at-risk for clinically significant mental health concerns and that identified cut-off scores should be used to prompt appropriate referrals. These findings are in line with recommendations from the National Athletic Trainer’s Association that in order to promote the health and well-being of student-athletes, universities should utilize empirically validated mental health screening instruments (Conley et al., 2014; Kroshus, 2016). Results of this study suggest scores over 32 on this measure are predictive of significant, and potentially referable, mental health concerns (T-score greater than 60 on the SCL-90 R); which is considered by the NCAA to be a crucial aspect of the screening process (NCAA Sport Science Institute, 2016). Independent athlete-type ROC analyses also support a cut-off score of 32. Although, this cutoff score was selected because it provided high levels of both sensitivity and specificity, higher or lower scores may be used if higher specificity or sensitivity, respectfully, is desired (cutoff scores are outlined in Table 7). In addition, the standard error of measurement was 4.69 on the MHDSIA and because the items may not be parallel, the SEM may underestimate how far the observed scores are from true scores. Due to its predictive capability, the MHDSIA may be useful in linking student-athletes to warranted mental health services who may have otherwise been missed. Along these lines, they may be more likely to seek treatment if they realize the effect improvements in mental health difficulties have on their performance (Gulliver et al., 2015; Gouttebarge et al., 2017).

In regards to feasibility, the results of this study suggest the MHDSIA may be cost-effectively implemented with athletes who are determined to be at-risk for mental health disorders by licensed mental health experts working with trained personnel (e.g., athletic trainers), or broadly implemented across teams, universities or leagues by athletic administrations or schools using confidentially-protected computer-based programs that are appropriately monitored by licensed mental health professionals. Administration of this scale may be completed in under 2 min and items appear to measure what they are intended to measure. The clinical utility of the MHDSIA is enhanced by cutoff scores that help to identify individuals who may benefit from a referral to mental health services.

Implementation of such strategies facilitates athletes’ awareness of mental health functioning outside of sports, potentially improving the pursuit of mental health care in athletes. Future outcome research might examine whether discussion of MHDSIA responses is more effective than discussion of responses to other general mental health screeners in the improvement of motivation for mental healthcare (Pinkerton et al., 1989; Donohue et al., 2016). The MHDSIA provides an opportunity to establish specific treatment goals by identifying elevations in performance interference in particular domains outside of sport. Supporting this contention, high specificity of treatment goals can help provide clarity concerning which specific interventions are likely to be beneficial (Miller and Rollnick, 2013).

Lastly, the sample size (259) was probably enough to yield a stable factor structure. However, a sample size of 300 is recommended when evaluating this type of data to prevent uninterpretable or unstable factor matrices (Tabachnick and Fidell, 2013). Future research is needed to confirm the factor structure that was found in the current sample.

## Data availability statement

The original contributions presented in the study are included in the article/supplementary material, further inquiries can be directed to the corresponding author.

## Ethics statement

The studies involving human participants were reviewed and approved by the University of Nevada, Las Vegas International Review Board. Written informed consent to participate in this study was provided by the participants.

## Author contributions

All authors listed have made a substantial, direct, and intellectual contribution to the work and approved it for publication.

## Funding

This research study was supported by a grant from the National Institutes of Health in the United States (National Institute on Drug Abuse award #1R01 DA031828). Publishing costs were funded by the University Libraries Open Article Fund and the College of Liberal Arts at the University of Nevada, Las Vegas. This study was funded by the National Institute on Drug Abuse; NIDA; 1 R01 DA031828.

## Conflict of interest

The authors declare that the research was conducted in the absence of any commercial or financial relationships that could be construed as a potential conflict of interest.

## Publisher’s note

All claims expressed in this article are solely those of the authors and do not necessarily represent those of their affiliated organizations, or those of the publisher, the editors and the reviewers. Any product that may be evaluated in this article, or claim that may be made by its manufacturer, is not guaranteed or endorsed by the publisher.

## Appendix

**MHDSIA**

Below is a list of things that sometimes occur with athletes during their lives outside of sports. Please circle the number that represents how often each of these things interferes with your life outside of sports.

Please use the following scale for items 1 through 14:

1 = *Never*, 2 = *Very Seldom*, 3 = *Seldom*, 4 = *Sometimes*, 5 = *Often*, 6 = *Very Often*, 7 = *Always.*

**Table:**

	How often does this interfere with your performance during your life outside of sports?
1. Too impulsive	1 2 3 4 5 6 7
2. Feeling depressed	1 2 3 4 5 6 7
3. Severe anxiety, panic attacks, doing senseless behavior repeatedly	1 2 3 4 5 6 7
4. Alcohol use	1 2 3 4 5 6 7
5. Drug use, or use of prescribed drugs more than a medical doctor’s recommendation	1 2 3 4 5 6 7
6. Difficulty maintaining weight at an acceptable level to me or others	1 2 3 4 5 6 7
7. Difficulty sleeping	1 2 3 4 5 6 7
8. Doing things that get me into trouble with others	1 2 3 4 5 6 7
9. Poor relationships with others	1 2 3 4 5 6 7
10. Tics or sudden and uncontrollable jerks of body parts	1 2 3 4 5 6 7
11. Hearing, smelling, or seeing things that others do not	1 2 3 4 5 6 7
12. Difficulties remembering things	1 2 3 4 5 6 7
13. Sudden mood swings	1 2 3 4 5 6 7
14. Sexual disorders (pain during sex, premature ejaculation, problems with arousal)	1 2 3 4 5 6 7
